# Semantic Topological Multi-Scale Part Network for Fine-Grained Visual Classification

**DOI:** 10.3390/jimaging12090407

**Published:** 2026-08-28

**Authors:** Xuerong Liu, Min Zhi, Yanjun Yin, Rula Sa

**Affiliations:** College of Computer Science and Technology, Inner Mongolia Normal University, Hohhot 010022, China; 20245017012@mails.imnu.edu.cn (X.L.);

**Keywords:** fine-grained visual classification, semantic part modeling, topological relationship learning, foundation model prior, multi-scale feature fusion

## Abstract

Fine-grained visual classification (FGVC) aims to distinguish highly similar subcategories, and its performance relies heavily on the accurate modeling of discriminative local parts and their structural relationships. However, existing Vision Transformer-based methods are susceptible to background noise interference, and the relationship modeling approach relying on explicit spatial coordinates struggles to maintain stable structural representations when targets undergo pose variations and non-rigid deformations. To address these issues, this paper proposes a Semantic Topology Part Network (STP-Net). First, a Prior-Guided Part Aggregator (PGA) is designed, which leverages the foreground prior provided by foundation models to guide discriminative part discovery, enhancing target region responses while suppressing background interference. Second, a Topology-Informed Semantic Graph Convolutional Network (TIS-GCN) is designed to dynamically construct topological relationships among parts in an implicit semantic space, achieving robust modeling against complex structural variations. Furthermore, a Semantic–Spatial Cross-Attention (SSCA) mechanism is introduced to establish bidirectional interaction between semantic relationships and spatial features, and combined with a Global-Context Adaptive Gating mechanism to accomplish multi-scale feature fusion. On four mainstream fine-grained visual classification benchmarks, namely CUB-200-2011, Stanford Cars, Stanford Dogs, and NABirds, the proposed model achieves Top-1 accuracies of 92.7%, 94.9%, 95.2%, and 92.3%, respectively. Comprehensive ablation studies and visualization analyses further validate the effectiveness of the proposed method in background suppression, structural relationship modeling, and discriminative feature learning.

## 1. Introduction

Fine-grained visual classification (FGVC) aims to distinguish subcategories within a broader object category (e.g., different species of birds or different models of cars) [1]. The fundamental challenge of FGVC stems from two inherent characteristics: minimal inter-class differences and substantial intra-class variations. To discriminate subtle inter-class differences (e.g., the specific curvature of a bird’s beak), the network must precisely localize discriminative local parts without being distracted by cluttered backgrounds. Meanwhile, to cope with significant intra-class variations caused by pose and viewpoint changes, the network must robustly model the structural relationships among these parts.

Although recent Vision Transformer-based fine-grained classification methods and part-based representation learning frameworks have significantly improved recognition performance through attention mechanisms, their core designs still face two fundamental limitations. First, background co-adaptation weakens inter-class discriminability. Due to the lack of explicit object-level inductive bias, the self-attention mechanism assigns high weights to visually salient background noise, causing the model to rely on spurious contextual clutter rather than mining the subtle yet genuine semantic parts that distinguish highly similar categories [2]. Second, existing relationship modeling methods universally rely on explicit geometric coordinates to construct part graph structures, such as Euclidean distance, relative angles, or polar coordinate relationships [3]. Although these methods can achieve favorable results under limited pose variations, absolute spatial coordinates undergo drastic fluctuations when targets experience significant non-rigid deformations, leading to unstable graph structures or even topological relationship collapse.

To address the aforementioned issues, this paper proposes a Semantic Topology Part Network (STP-Net). The core idea is to perform discriminative part discovery guided by foundation model priors and model structural relationships among parts in an implicit semantic space rather than relying on fragile geometric coordinates. By learning adaptive semantic relationships among part representations, STP-Net provides a more robust structural representation under pose variations and non-rigid deformations. Specifically, a Prior-Guided Part Aggregator (PGA) is first designed, which leverages semantic masks generated by foundation models (e.g., SAM [4]) as spatial priors during the training phase, guiding the network to focus on target foreground regions and aggregate discriminative parts, thereby enhancing feature quality. Second, a Topology-Informed Semantic Graph Convolutional Network (TIS-GCN) is proposed to dynamically construct part relationship graphs in a high-dimensional semantic space, achieving structural modeling that is robust to pose variations and non-rigid deformations. Then, a Semantic–Spatial Cross-Attention module (SSCA) is designed to facilitate effective interaction between semantic topological information and multi-scale spatial features, thereby generating a global representation that is both discriminative and structure-aware. Finally, an auxiliary deep supervision strategy is introduced to enhance optimization stability during hierarchical feature learning. Through the synergistic effect of the above modules, STP-Net is capable of learning more robust fine-grained representations and significantly improving fine-grained visual classification performance.

The main contributions of this work are summarized as follows:

1. A fine-grained visual classification framework, STP-Net, is proposed that integrates foundation model priors with semantic topological reasoning. By unifying discriminative part discovery and topologically invariant relationship modeling, it effectively alleviates background interference and structural instability, enhancing fine-grained recognition capability in complex scenarios.
2. A PGA is proposed that leverages foreground masks generated by foundation models as semantic priors during training to guide discriminative part aggregation, enhancing foreground awareness.
3. A topologically invariant relationship modeling mechanism composed of TIS-GCN and SSCA is proposed, which achieves robust structural representation learning against non-rigid deformations by dynamically constructing adjacency relationships in the semantic space.
4. Extensive experiments are conducted on four fine-grained benchmark datasets, namely CUB-200-2011, Stanford Cars, Stanford Dogs, and NABirds, achieving Top-1 classification accuracies of 92.7%, 94.9%, 95.2%, and 92.3%, respectively. Ablation studies and visualization analyses further validate the effectiveness and interpretability of each module design.

## 2. Related Work

### 2.1. Discriminative Part Learning

The core of fine-grained visual classification lies in mining local discriminative features capable of distinguishing highly similar subcategories. Early studies typically relied on strongly supervised information such as bounding boxes, keypoints, and part annotations [5,6,7] to achieve precise localization. Such methods are highly dependent on expensive manual annotations, limiting their practical applicability. With the development of weakly supervised learning, research focus has gradually shifted toward discriminative region discovery without additional part annotations [8,9,10,11,12,13,14]. In recent years, Vision Transformer (ViT) has achieved remarkable progress in the FGVC field owing to its global self-attention mechanism. TransFG [15] achieves fine-grained recognition by selecting key tokens with high attention responses; IELT [16] enhances multi-layer feature representation through internal ensemble learning; LDH-ViT [17] promotes discriminative region discovery via a local hiding strategy; DAVT [18] combines data augmentation with the Transformer architecture to improve model generalization; FET-FGVC [19] further strengthens fine-grained representation learning through a feature enhancement mechanism. Meanwhile, recent works such as ACC-ViT [20], MP-FGVC [21], and LGTF [22] continue to improve the performance of Transformers in fine-grained recognition tasks through cross-layer interaction, multi-granularity modeling, and local–global collaborative learning.

Although the aforementioned methods can effectively discover discriminative regions, most of them primarily rely on attention responses for part selection, lacking an explicit foreground constraint mechanism. When statistical correlations exist between the background and category labels, the model is prone to background co-adaptation, erroneously adopting background context as the basis for classification, thereby compromising the generalization performance of the model.

### 2.2. Part Structural Relationship Modeling

The structural relationships among parts also constitute an important basis for fine-grained visual classification. To enhance the structural modeling capability of fine-grained targets, a substantial body of research has begun incorporating Graph Neural Networks (GNNs) and Graph Convolutional Networks (GCNs) into FGVC tasks. SIM-Trans [3] constructs structural graphs through explicit geometric relationships to enhance part interaction; SFEtrans [23] strengthens information propagation among parts via a spatial feature enhancement module; MAS-ViT [24] further establishes structural constraints by combining polar coordinate relationships with cross-layer feature fusion. In addition, some studies attempt to leverage structural priors [25] and graph relational reasoning [26] to enhance the model’s adaptability to complex pose variations.

However, most existing methods rely on explicit spatial information such as Euclidean distance, relative position, or polar coordinates to construct relationship graphs. When targets undergo significant pose changes or non-rigid deformations, absolute spatial coordinates change substantially, leading to unstable graph structures or even failure in relationship modeling. This is particularly evident in datasets featuring objects with complex pose variations, such as birds and dogs, where coordinate-based relationship modeling often struggles to maintain structural consistency.

Different from these methods that mainly rely on explicit spatial relationships or attention-based feature interaction for part modeling, STP-Net introduces semantic topology reasoning to learn adaptive relationships among discriminative parts in an implicit semantic space, reducing the dependence on unstable geometric coordinates under pose variations and deformations.

### 2.3. Foundation Model Prior Guidance

In recent years, the Segment Anything Model (SAM) [4], trained on large-scale mask data, has demonstrated the ability to generate high-quality object masks under zero-shot conditions, providing reliable semantic priors for target region localization, background suppression, and discriminative region discovery. This offers a novel solution for fine-grained target localization and part discovery. Some studies leverage SAM-generated object masks to assist in discriminative region localization [27]; others combine semantic knowledge from vision–language models to enhance fine-grained category representation [28]; recent research also attempts to utilize structural priors generated by foundation models to guide local feature learning, thereby improving fine-grained recognition performance [29].

Although foundation models can significantly enhance fine-grained feature learning capabilities, most existing methods require continuous invocation of foundation models during the inference stage, resulting in additional storage overhead and inference latency.

## 3. Proposed Method

### 3.1. Overall Architecture

**Figure 1:**
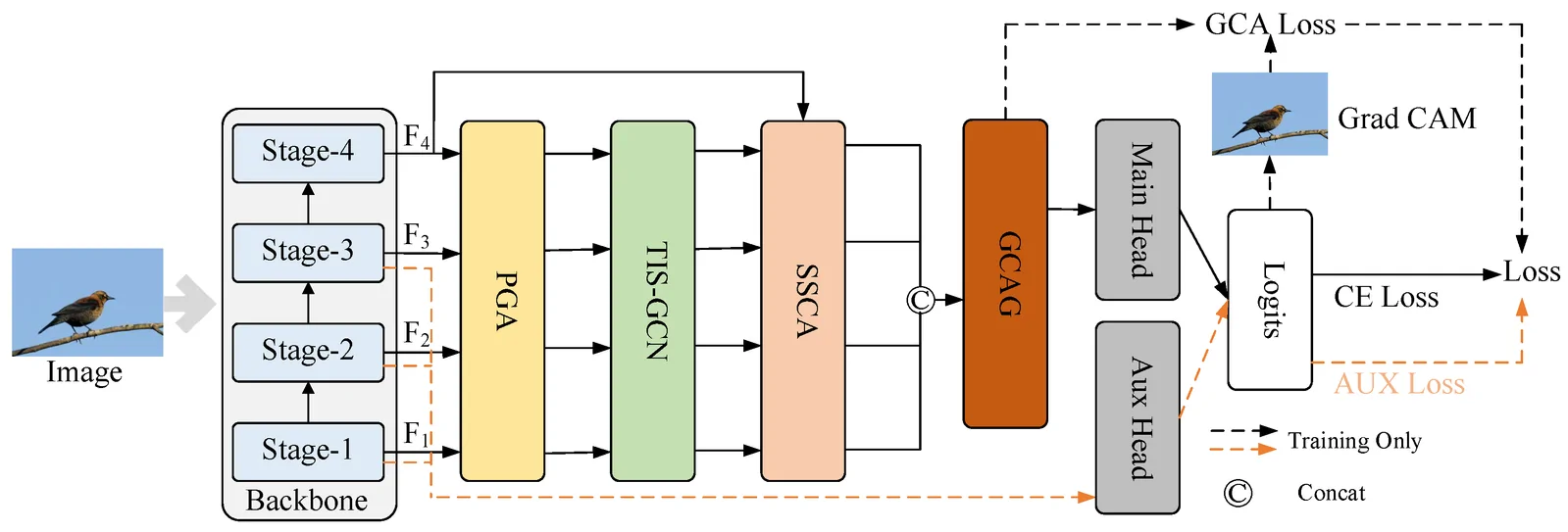
Overall architecture of STP-Net based on the Swin Transformer backbone.

The overall architecture of the proposed STP-Net is illustrated in Figure 1. STP-Net is a fine-grained visual classification framework built upon a Swin Transformer backbone. In this framework, the backbone network is responsible for extracting hierarchical multi-scale visual features, while the proposed modules further perform semantic-guided part representation and structural relationship modeling. Specifically, STP-Net adopts a hierarchical multi-scale feature modeling strategy aimed at fully mining fine-grained discriminative information from different semantic granularities. Given an input image $I\in R^{3\times H\times W}$, feature representations ${\left\{F_{s}\right\}}_{s=1}^{4}$ are extracted through a hierarchical backbone network at four consecutive stages, where $F_{s}\in R^{H_{s}\times W_{s}\times C_{s}}$. Features at different levels retain complementary information ranging from local textures to high-level semantics, providing a multi-scale foundation for subsequent semantic part aggregation and topological relationship reasoning.

The multi-stage features are fed into a parallel progressive discriminative enhancement path composed of PGA, TIS-GCN, and SSCA. First, the Prior-Guided Part Aggregator (PGA) adaptively generates semantic part tokens on features at each scale, effectively reducing the influence of irrelevant background responses with the aid of foundation model priors, thereby improving the response quality of key local regions. Subsequently, the Topology-Informed Semantic Graph Convolution (TIS-GCN) explicitly models higher-order relationships among part tokens in the semantic graph space, enhancing structural discriminability among fine-grained categories through relationship propagation. Finally, Semantic–Spatial Cross-Attention (SSCA) further establishes bidirectional interaction between semantic part representations and backbone spatial features, remapping abstract semantic relationships back into the spatial context to form enhanced representations that possess both structural awareness and spatial localization capabilities.

To further fuse complementary discriminative information from different levels, the outputs of each stage are first concatenated along the channel dimension and then fed into a Global-Context Adaptive Gating (GCAG) module, which achieves selective enhancement of cross-scale features through a global context-aware channel recalibration mechanism, thereby obtaining a unified and more discriminative global representation.

During the supervised optimization stage, the network employs a dual-head joint supervision strategy. The main classification head (Main Head) outputs the final category prediction logits based on the fused features; the auxiliary classification head (Aux Head) imposes auxiliary supervision on intermediate layer representations to alleviate gradient attenuation during deep network training and promote discriminative feature learning in early layers. The overall training objective consists of three components: the main-classification cross-entropy loss $L_{\mathrm{CE}}$, the auxiliary supervision loss $L_{\mathrm{AUX}}$, and the gradient-attention consistency loss $L_{\mathrm{GCA}}$ [30]. Specifically, $L_{\mathrm{GCA}}$ constrains the consistency between the network’s attention regions and the Grad-CAM salient responses, guiding the model to focus on more semantically interpretable discriminative regions. It should be emphasized that $L_{\mathrm{GCA}}$ and $L_{\mathrm{AUX}}$ are only enabled during the training phase.

### 3.2. Prior-Guided Part Aggregator

In fine-grained visual classification, discriminative regions are usually distributed in small object parts, making accurate part discovery challenging under weak supervision. Although Transformer-based models can capture informative regions through self-attention, the learned attention maps may be affected by irrelevant background patterns due to the absence of explicit semantic guidance. Therefore, the model may fail to identify truly discriminative parts and learn unreliable representations.

**Figure 2:**
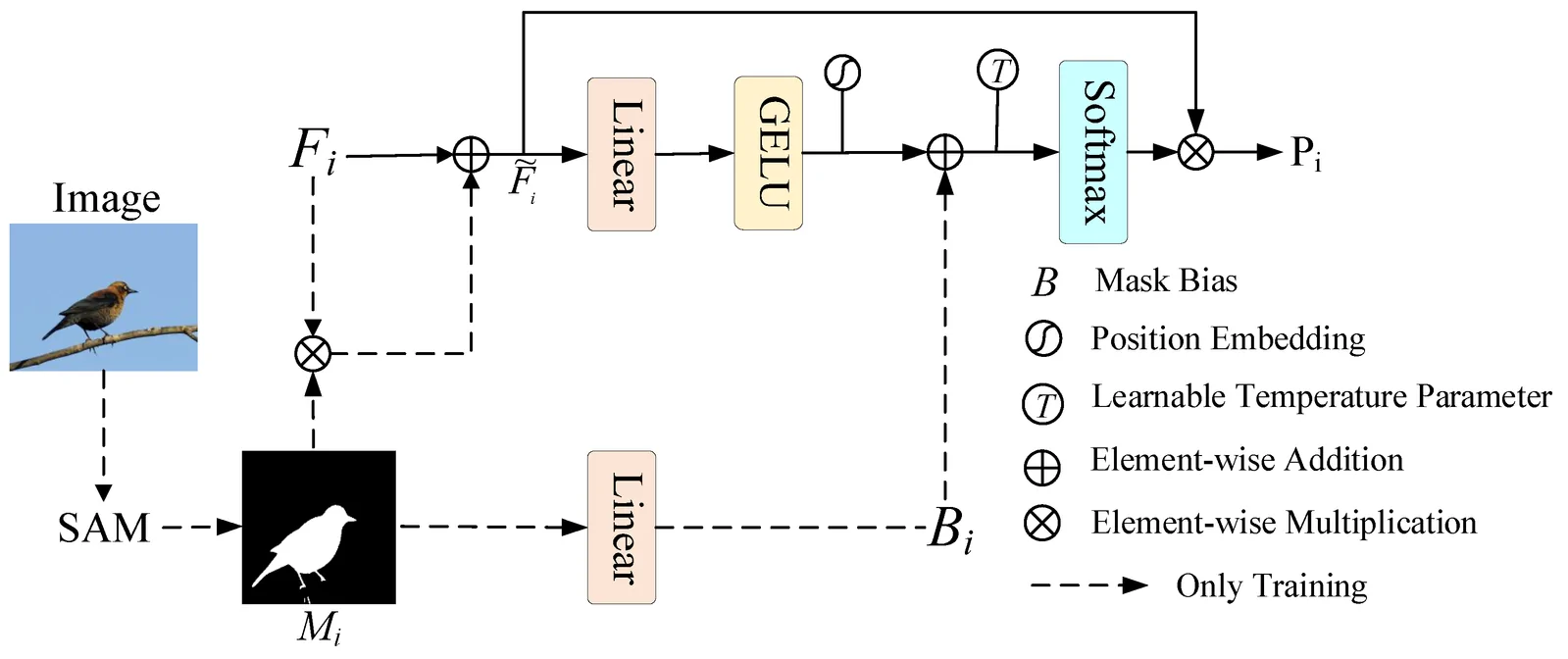
Prior-Guided Part Aggregator.

To address this issue, we propose a Prior-Guided Part Aggregator (PGA) module, which introduces foreground semantic guidance into the part aggregation process. Different from conventional approaches that rely only on visual responses, PGA incorporates foreground priors to constrain the learning of part representations, enabling the model to focus more on semantically meaningful regions. The architecture of the PGA module is illustrated in Figure 2.

It should be clarified that PGA is a newly proposed module in STP-Net and is not a component of the Segment Anything Model (SAM). In this work, SAM is only adopted as an auxiliary prior generator during the training stage to provide foreground masks. These masks serve as semantic guidance for optimizing PGA, while the architecture and inference process of PGA are independent of SAM. After training, SAM is completely removed, and the learned PGA module performs part aggregation without additional external assistance.

Given the hierarchical feature representation extracted from the backbone:

(1) $$F_{i}\in R^{H_{i}\times W_{i}\times C_{i}}$$

where $H_{i}$, $W_{i}$, and $C_{i}$ denote the height, width, and channel dimensions of the feature map at the *i*-th stage, respectively.

During training, the foreground prior generated by SAM is resized to match the spatial resolution of $F_{i}$, represented as:

(2) $$M_{i}\in R^{H_{i}\times W_{i}}$$

where $M_{i}$ provides semantic information for distinguishing foreground regions from irrelevant background areas.

To obtain target-aware representations, we first enhance the original features by integrating the foreground prior during the training stage:

(3) $${\tilde{F}}_{i}=\left\{\begin{array}{cc} F_{i}+F_{i}⊙\left(\alpha M_{i}\right), & \mathrm{Training}\\ F_{i}, & \mathrm{Inference} \end{array}\right.\in R^{H_{i}\times W_{i}\times C_{i}}$$

where ⊙ denotes element-wise multiplication, $\alpha \in R^{H_{i}\times W_{i}\times 1}$ represents the learnable coefficient controlling the influence of the foreground prior, and ${\tilde{F}}_{i}$ denotes the target-aware enhanced feature. During the training stage, the foreground mask $M_{i}$ generated by SAM is used to enhance target regions, thereby obtaining more target-aware representations. During the inference stage, the model no longer relies on SAM and directly uses the original visual feature $F_{i}$.

Second, the foreground prior is further transformed into a semantic bias to guide part attention learning:

(4) $$B_{i}=\left\{\begin{array}{cc} Linear(M_{i}), & Training\\ 0, & Inference \end{array}\right.$$

where $B_{i}$ denotes the prior bias generated from the foreground mask, which is a learnable bias term.

Subsequently, the enhanced feature representation is projected into the part feature space by combining linear transformation, GELU activation, positional embedding, and prior bias:

(5) $$S_{i}=\left\{\begin{array}{cc} \mathrm{GELU}\left(\mathrm{Linear}\left({\tilde{F}}_{i}\right)\right)+E_{pos}+B_{i}, & Training\\ \mathrm{GELU}\left(\mathrm{Linear}\left({\tilde{F}}_{i}\right)\right)+E_{pos}, & Inference \end{array}\right.$$

where $N_{i}=H_{i}\times W_{i}$, $E_{\mathrm{pos}}\in R^{N_{i}\times C_{i}}$ represents positional encoding for preserving spatial information, and $B_{i}\in R^{N_{i}}$ is broadcast from the previous bias. Compared with conventional attention mechanisms that depend solely on visual features, this design introduces explicit semantic constraints during part discovery, reducing the influence of background noise.

To obtain adaptive part selection, a learnable temperature parameter *T* is introduced to regulate the attention distribution:

(6) $$A_{i}=\mathrm{Softmax}\left(\frac{S_{i}}{T}\right)\in R^{K_{i}\times N_{i}}$$

where $A_{i}$ denotes the generated attention weights, T∈R is a learnable scalar temperature parameter. The temperature parameter *T* controls the sharpness of the attention distribution. A larger value of *T* produces a smoother attention distribution by reducing the differences among response scores, while a smaller value of *T* results in a sharper distribution that concentrates attention on more discriminative regions. During training, *T* is adaptively optimized to balance global feature coverage and discriminative part localization.

Finally, the attention weights are applied to the original feature representation to obtain the aggregated part features:

(7) $$P_{i}=A_{i}⊙F_{i}\in R^{K_{i}\times C_{i}}$$

where $P_{i}$ represents the discriminative part representation generated by the *i*-th stage, and $F_{i}\in R^{N_{i}\times C_{i}}$ is the reshaped original feature map. The number of parts is defined as $K_{i}=C_{i}/2$, where $C_{i}$ denotes the channel dimension of the feature map at the *i*-th stage.

It should be noted that $K_{i}$ represents the number of learnable part prototypes rather than the number of spatial tokens. Therefore, $K_{i}$ is not restricted by the spatial resolution of the feature map. Each part token is adaptively generated by aggregating spatial features through the attention mechanism, allowing multiple semantic parts to be discovered from shared feature representations, rather than simply assigning one token to one spatial location.

Since the attention weights integrate visual features, spatial information, and foreground semantic guidance, PGA can generate more reliable part representations while reducing background interference.

### 3.3. Topology-Informed Semantic Graph Convolution

**Figure 3:**
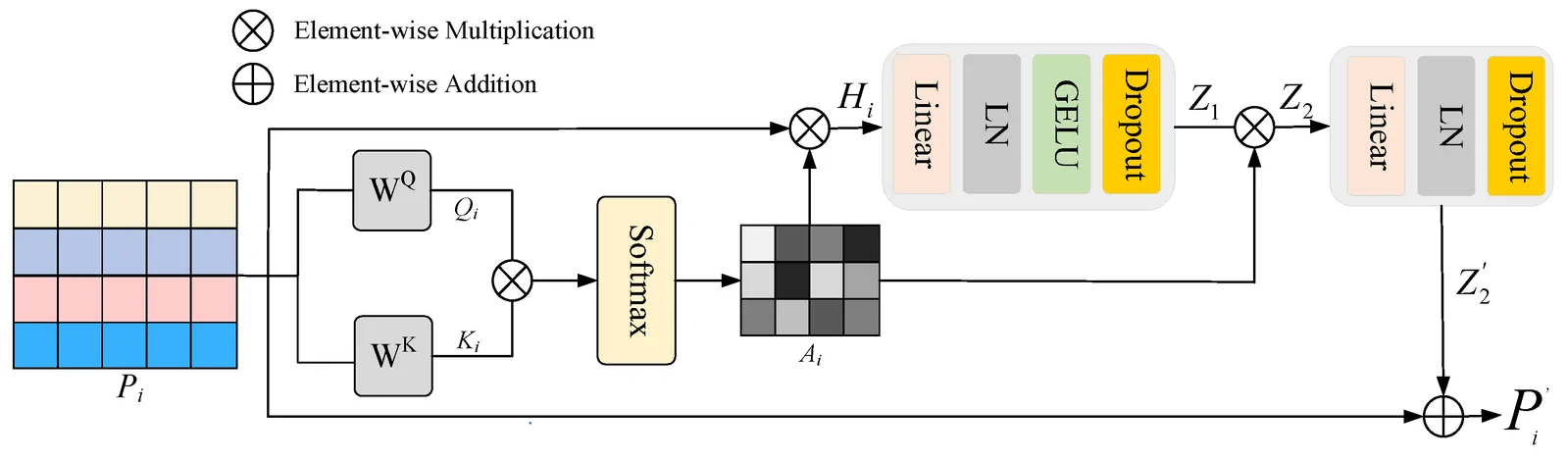
Topology-Informed Semantic Graph Convolution.

Fine-grained visual classification requires not only discovering discriminative local parts but also effectively modeling the structural relationships among parts. However, existing graph structure-based methods typically rely on explicit geometric information such as Euclidean distance, relative coordinates, or polar coordinates to construct adjacency relationships. When targets undergo non-rigid deformations, the physical distances between semantically identical parts often change significantly, leading to unstable graph topological structures and limiting the relationship modeling capability. To this end, this paper proposes the Topology-Informed Semantic Graph Convolution (TIS-GCN), as shown in Figure 3, which dynamically constructs adjacency relationships in the semantic feature space to achieve structural modeling that is more robust to pose variations and deformation perturbations.

Let the part feature representation output by the PGA module be $P_{i}\in R^{K\times C}$, where *K* denotes the number of parts and *C* denotes the feature dimension. Unlike traditional GCN that relies on pre-defined spatial topologies, TIS-GCN directly leverages semantic similarity among parts to construct dynamic graph structures.

First, query features and key features are generated through two independent linear mappings:

(8) $$Q_{i}=P_{i}W^{Q}\in R^{K\times d},\;K_{i}=P_{i}W^{K}\in R^{K\times d}$$

where $W^{Q}\in R^{C\times d}$, $W^{K}\in R^{C\times d}$ denote the query and key mapping matrices, respectively, and *d* denotes the embedding dimension.

Subsequently, the semantic correlation among parts is computed to construct the dynamic adjacency matrix:

(9) $$A_{i}=\mathrm{Softmax}\left(\frac{Q_{i}K_{i}^{⊤}}{\sqrt{d}}\right)$$

where $A_{i}\in R^{K\times K}$ denotes the learned semantic topological structure. Unlike methods that construct adjacency relationships based on Euclidean distance, the connection strength in Equation (9) is entirely determined by the semantic similarity among parts. Therefore, even when targets undergo significant pose variations or non-rigid deformations, as long as the parts maintain similar semantic attributes, their topological relationships remain stable, thereby improving the robustness of structural modeling.

After obtaining the dynamic graph structure, the adjacency matrix is used to perform relationship propagation on the part features:

(10) $$H_{i}=A_{i}P_{i}\in R^{K\times C}$$

where $H_{i}$ denotes the neighborhood-aggregated part features. Through neighborhood information aggregation, each part node not only retains its own local discriminative information but also perceives the contextual structural relationships associated with it.

Subsequently, a feed-forward mapping module is employed to further enhance the structural representation capability:

(11) $$Z_{1}=\mathrm{Dropout}\left(\mathrm{GELU}\left(\mathrm{LN}\left(\mathrm{Linear}\left(H_{i}\right)\right)\right)\right)\in R^{K\times C}$$

To prevent the original discriminative information from being over-smoothed during graph propagation while further strengthening the structural relationship modeling capability, a residual enhancement mechanism is introduced:

(12) $$Z_{2}=A_{i}Z_{1}\in R^{K\times C}$$

Subsequently, the structural features are further refined through linear transformation and normalization operations:

(13) $$Z_{2}^{\prime }=\mathrm{Dropout}\left(\mathrm{LN}\left(\mathrm{Linear}\left(Z_{2}\right)\right)\right)\in R^{K\times C}$$

Inspired by the LayerScale concept, a learnable scaling parameter γ is introduced to control the intensity of structural information injection, yielding the enhanced part representation:

(14) $$P_{i}^{\prime }=P_{i}+\gamma Z_{2}^{\prime }\in R^{K\times C}$$

Through the above process, TIS-GCN transfers graph structure construction from the fragile geometric coordinate space to the more stable semantic feature space, achieving topologically invariant relationship modeling. While preserving local discriminative information, the network effectively captures higher-order structural dependencies among parts, thereby enhancing the model’s robustness to complex pose variations, partial occlusions, and intra-class variations, and providing more reliable structural representations for the subsequent Semantic–Spatial Cross-Attention module.

### 3.4. Semantic–Spatial Cross-Attention

**Figure 4:**
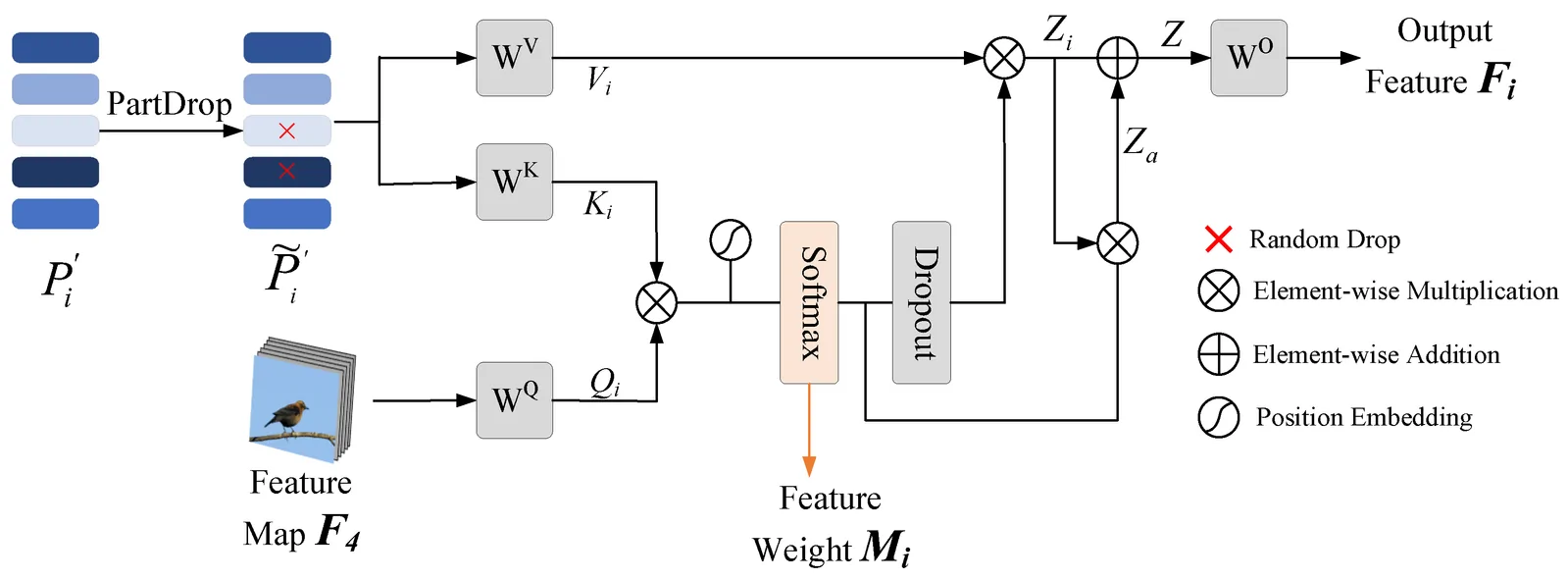
Semantic–Spatial Cross-Attention.

After discriminative part discovery by the PGA module and topological relationship modeling by the TIS-GCN module, the network has obtained part representations containing rich semantic structural information. However, these part features primarily reside in the semantic relationship space and lack sufficient interaction with the original visual features. If the graph-reasoned part features are directly used for classification, it tends to cause a semantic disconnection between local structural information and global visual context, thereby limiting the final classification performance. To this end, this paper designs the Semantic–Spatial Cross-Attention module (SSCA), as shown in Figure 4, which achieves deep fusion of structural semantics and spatial visual features through a cross-attention mechanism, thereby constructing a global representation that is both discriminative and structure-aware.

Let the structure-enhanced part features output by TIS-GCN be $P_{i}^{\prime }\in R^{K\times C}$, where *K* denotes the number of parts and *C* denotes the feature dimension. Meanwhile, the feature map output by the last stage of the backbone network is denoted as $F_{4}\in R^{N\times C}$, where $N=H_{4}\times W_{4}$ denotes the number of spatial tokens.

To prevent the model from chronically relying on fixed part regions and to improve generalization capability, a PartDrop strategy is first introduced for the part features. During the training phase, a portion of part nodes is randomly dropped, thereby forcing the model to learn richer discriminative patterns:

(15) $${\hat{P}}_{i}^{\prime }=\mathrm{PartDrop}\left(P_{i}^{\prime }\right)\in R^{K\times C}$$

Subsequently, linear mappings are used to construct the query, key, and value representations:

(16) $$Q_{i}=F_{4}W^{Q}\in R^{N\times d},\;K_{i}={\hat{P}}_{i}^{\prime }W^{K}\in R^{K\times d},\;V_{i}={\hat{P}}_{i}^{\prime }W^{V}\in R^{K\times d}$$

where $W^{Q}\in R^{C\times d}$, $W^{K}\in R^{C\times d}$, and $W^{V}\in R^{C\times d}$ are learnable projection matrices, and *d* denotes the embedding dimension.

To preserve spatial position information, positional encoding $E_{\mathrm{pos}}\in R^{N\times d}$ is introduced into the query features, and the cross-attention weights between spatial features and part features are computed:

(17) $$A_{i}=\mathrm{Softmax}\left(\frac{Q_{i}K_{i}^{⊤}}{\sqrt{d}}+E_{\mathrm{pos}}\right)\in R^{N\times K}$$

where $A_{i}$ denotes the association weight matrix between spatial tokens and semantic parts. Through this weight matrix, information interaction between the visual space and the semantic structure space is achieved. The attention weights are then used to perform weighted aggregation of the part features:

(18) $$Z_{i}=\mathrm{Dropout}\left(A_{i}\right)V_{i}\in R^{N\times d}$$

where $Z_{i}$ denotes the spatially enhanced features after fusing structural relationships.

To further emphasize high-confidence response regions and alleviate the influence of background interference, we introduce an attention-guided feature recalibration strategy. Specifically, instead of relying solely on the primary attention distribution, a secondary normalization is performed on the attention scores $A_{i}$ across the semantic part dimension. By averaging over all attention heads and accumulating the responses from all *K* semantic parts, we generate a global feature weight vector $G_{i}$ that characterizes the importance of each spatial token:

(19) $$G_{i}=\sum_{k=1}^{K}{\mathrm{Mean}}_{h}\left({\mathrm{Softmax}}_{2}\left(A_{i,h,n,k}\right)\right)\in R^{N}$$

where ${\mathrm{Softmax}}_{2}$ denotes the secondary normalization. This operation effectively captures the cumulative contribution of various semantic parts to each spatial location.

Subsequently, the aggregated features $Z_{i}$ are adaptively modulated by the weight vector $G_{i}$. Utilizing a learnable scaling parameter γ∈R (corresponding to weights_scale in implementation), the model applies a nonlinear gain to the discriminative regions:

(20) $$Z_{i}^{\prime }=Z_{i}⊙\left(1+G_{i}\cdot \gamma \right)\in R^{N\times d}$$

where ⊙ denotes element-wise multiplication. This enhancement ensures that spatial tokens with strong semantic correlations receive higher activation, thereby improving the discriminability of the features in cluttered environments.

Finally, the structure-aware global representation ${\hat{F}}_{i}$ is obtained through the output projection mapping:

(21) $${\hat{F}}_{i}=Z_{i}^{\prime }W^{O}\in R^{N\times C}$$

where $W^{O}\in R^{d\times C}$ denotes the output projection matrix, and ${\hat{F}}_{i}$ denotes the final structure-aware spatial features. By integrating this dual-normalization and self-enhancement process, the SSCA module effectively bridges the semantic relationship space and the visual spatial context, allowing the network to simultaneously perceive fine-grained local details and higher-order topological structures.

### 3.5. Hierarchical Fusion and Multi-Objective Supervision

After completing semantic part modeling and semantic–spatial interaction, how to effectively integrate multi-scale information and stably optimize the entire network becomes a critical issue. To this end, a unified hierarchical fusion and multi-objective supervision framework is designed to enable the model to strike a balance between global semantic consistency and local discriminative capability.

Specifically, the relationship-aware features output by the SSCA module at stage *s* are denoted as $X_{s}\in R^{N\times C}$ (s=1,2,3,4), where $N=H_{4}\times W_{4}$ denotes the number of spatial tokens aligned to the resolution of the deepest stage (Stage 4). Thanks to the cross-attention design in SSCA, where $F_{4}$ acts as the query and the multi-scale part tokens serve as keys/values, all stage outputs $X_{s}$ are projected onto the unified spatial grid of $F_{4}$ before fusion. Consequently, these features can be directly concatenated along the channel dimension to form the fused representation $X_{\mathrm{cat}}=\left[X_{1};X_{2};X_{3};X_{4}\right]\in R^{N\times 4C}$. This concatenation operation preserves complementary information ranging from fine-grained textures to high-level semantics at different scales. To address the redundancy introduced by direct concatenation, a Global-Context Adaptive Gating (GCAG) mechanism is introduced to dynamically recalibrate the multi-scale features. Specifically, the global context descriptor $z\in R^{4C}$ is extracted through global average pooling, and a channel-level gating vector $g\in R^{4C}$ is generated through a two-layer nonlinear mapping:

(22) $$g=\sigma \left(W_{2}\;\mathrm{GELU}\left(W_{1}z\right)\right)\in R^{4C}$$

where $W_{1}\in R^{4C\times 2C}$, $W_{2}\in R^{2C\times 4C}$ denote the learnable weight matrices, and σ denotes the Sigmoid function. The final fused features are obtained through channel-wise adjustment:

(23) $$X_{\mathrm{fused}}=X_{\mathrm{cat}}⊙g\in R^{N\times 4C}$$

where ⊙ denotes channel-wise multiplication, and $X_{\mathrm{fused}}$ denotes the selectively enhanced fused features. This mechanism enables the network to adaptively select the importance of features at different scales based on the input content, thereby achieving more robust cross-scale semantic fusion.

On this basis, the fused features are fed into the main classification head to generate the final prediction results $y_{\mathrm{main}}\in R^{C_{\mathrm{cls}}}$. Meanwhile, to alleviate the insufficient learning of low-level features in deep networks, a hierarchical deep supervision strategy is introduced. Auxiliary classification branches are constructed at intermediate stages (s∈{1,2,3}) of the backbone network to supervise the corresponding level features:

(24) $$y_{\mathrm{aux}}^{\left(s\right)}=f_{s}\left(X_{s}\right)\in R^{C_{\mathrm{cls}}}$$

where $f_{s}$ denotes the classification head at the *s*-th stage, and $C_{\mathrm{cls}}$ denotes the number of categories. These auxiliary supervisions provide direct gradient signals for shallow features, effectively alleviating the gradient attenuation problem and promoting the full learning of bottom-level textures and local structural information, thereby enhancing the overall feature representation capability.

To further enhance the model’s interpretability and discriminative consistency, a gradient-attention consistency constraint is introduced. Specifically, the feature weight *M* generated by the SSCA module is regarded as the explicitly learned attention distribution, while the Grad-CAM saliency map *G* computed from backpropagation reflects the implicit gradient attribution information. By minimizing the discrepancy between them:

(25) $$L_{\mathrm{GCA}}=\frac{1}{B}\sum_{b=1}^{B}{∥M_{b}-G_{b}∥}_{2}^{2}$$

where *B* denotes the batch size, the model is constrained to maintain consistency between “explicit attention” and “true discriminative evidence”, thereby avoiding attention to irrelevant regions and improving the model’s interpretability and robustness.

Finally, the entire network is trained through multi-objective joint optimization:

(26) $$L_{\mathrm{total}}=L_{\mathrm{CE}}\left(y,y_{\mathrm{main}}\right)+\lambda_{\mathrm{aux}}\sum_{s=1}^{3}L_{\mathrm{CE}}\left(y,y_{\mathrm{aux}}^{\left(s\right)}\right)+\lambda_{\mathrm{gca}}L_{\mathrm{GCA}}$$

where $L_{\mathrm{CE}}$ denotes the cross-entropy loss with label smoothing, and $\lambda_{\mathrm{aux}}$ and $\lambda_{\mathrm{gca}}$ are balancing coefficients. This joint optimization strategy enables the model to achieve synergistic improvement among classification performance, structural modeling capability, and attention consistency.

## 4. Experiments

### 4.1. Datasets and Experimental Setup

STP-Net is evaluated on four standard FGVC benchmark datasets, as summarized in Table 1. The official train/test splits are followed, and Top-1 accuracy is adopted as the primary evaluation metric. CUB-200-2011 contains 11,788 bird images from 200 bird species, where the main challenges come from subtle inter-class differences, viewpoint changes, and complex backgrounds. Stanford Cars consists of 16,185 images from 196 car categories, focusing on distinguishing visually similar vehicle models. Stanford Dogs contains 20,580 images from 120 dog breeds, exhibiting large intra-class variations caused by pose, illumination, and appearance changes. NABirds is a large-scale bird dataset containing 48,562 images from 555 species, providing more challenging scenarios with greater category diversity and background complexity.

#### 4.1.1. Network Architecture and Hyperparameters

Swin-Base [35] pre-trained on ImageNet-21K is adopted as the multi-scale backbone network. All input images are uniformly resized to 384×384. In the PGA module, the Segment Anything Model 2 (SAM 2) Base Plus version (configuration: sam2_hiera_b+) is employed to generate foreground priors. The part sampling ratio is fixed at r=2. Given the channel dimensions $C_{s}\in \left\{128,256,512,1024\right\}$ at each level, the number of semantic parts discovered at each stage is computed as $K_{s}=C_{s}/r$, yielding {64,128,256,512} tokens, respectively. The sampling temperature parameter *T* is initially set to 1.2 and optimized end-to-end during training. In TIS-GCN, the implicit subspace dimension is $d_{\mathrm{sub}}=C/2$, message passing is performed through a two-layer GCN, and the LayerScale parameter γ is initialized to $10^{-4}$. For SSCA, the number of attention heads is set to H=32. The random part dropout (PartDrop) rate ρ is set to 0.4 on CUB, Cars, and Dogs, and 0.2 on NABirds. To prevent degradation of low-level features, the loss weights for auxiliary deep supervision (ADS) and gradient-attention consistency (GAC) are set to $\lambda_{\mathrm{aux}}=0.35$ and $\lambda_{\mathrm{gca}}=0.05$, respectively.

#### 4.1.2. Training Configuration

All models are implemented in PyTorch 2.8.0. The AdamW optimizer is employed for training (except for CUB and NABirds, which use SGD with a momentum of 0.9), with a weight decay of $1\times 10^{-8}$. A cosine annealing scheduler with 5 epochs of linear warmup is utilized. The training duration and learning rate vary across datasets: Stanford Cars and CUB-200-2011 are trained for 50 epochs with learning rates of $8\times 10^{-4}$ and $8\times 10^{-3}$, respectively. Stanford Dogs and NABirds follow the default 100 epochs with learning rates of $8\times 10^{-5}$ and $16\times 10^{-3}$, respectively. All experiments are conducted on a single NVIDIA RTX 4090 GPU. The training batch size is fixed at 16, yielding a global batch size of 16. Gradient clipping with a maximum norm of 10.0 is applied for CUB and NABirds to stabilize training.

### 4.2. Comparison with State-of-the-Art Methods

To validate the effectiveness of the proposed STP-Net, we conduct comprehensive comparisons against representative fine-grained visual classification methods, encompassing both conventional CNN-based approaches and recent Transformer-based models. Table 2 reports the Top-1 accuracy on four benchmarks, including CUB-200-2011, Stanford Cars, Stanford Dogs, and NABirds. It is important to clarify that the Segment Anything Model is employed solely as an external off-the-shelf tool for generating object masks during data preprocessing, and is not integrated into the trainable architecture of STP-Net. Consequently, the parameters and computational cost of SAM are excluded from all complexity statistics reported for our method. Additionally, we provide a computational complexity analysis in terms of FLOPs and parameters for Transformer-based competitors.

STP-Net achieves the best performance on three out of four datasets, obtaining 92.7%, 95.2%, and 92.3% Top-1 accuracy on CUB-200-2011, Stanford Dogs, and NABirds, respectively. These results demonstrate that the proposed semantic topology-based part modeling framework generalizes well across diverse fine-grained recognition scenarios. On CUB-200-2011 and NABirds, STP-Net outperforms TransFG by 1.0% and 1.3%, respectively, and surpasses ACC-ViT by 0.9% on NABirds. Bird recognition is particularly challenging due to complex backgrounds, large viewpoint variations, and frequent occlusions. The consistent improvements indicate that the Prior-Guided Aggregation module effectively leverages semantic priors to guide discriminative part discovery, while the Topology-Informed Spatial Graph Convolutional Network further enhances the modeling of semantic relationships among parts, yielding more robust avian representations.

On Stanford Dogs, STP-Net achieves 95.2% Top-1 accuracy, outperforming LDH-ViT and ACC-ViT by margins of 2.1% and 2.3%, respectively. Dog categories exhibit substantial intra-class variations caused by pose changes, deformations, and appearance diversity. Unlike methods that rely on fixed spatial layouts, STP-Net constructs adaptive semantic topological relationships among parts via the Topology-Informed Spatial Graph Convolutional Network, enabling stable structural representations under complex pose variations.

For the Stanford Cars dataset, STP-Net achieves a competitive accuracy of 94.9%, which is slightly lower than the 95.4% reported by P2P-Net. This marginal gap may be related to the different structural modeling strategies and design objectives of the two methods. Specifically, P2P-Net is designed to explicitly exploit geometric relationships among vehicle components, while STP-Net focuses on learning semantic topological relationships among discriminative parts. Nevertheless, STP-Net still surpasses several Transformer-based counterparts, including TransFG and LDH-ViT, demonstrating that semantic topology modeling effectively captures discriminative structural information across diverse fine-grained recognition scenarios.

We further analyze the computational overhead of STP-Net. As shown in Table 2, our method requires only 49.5 G FLOPs, a value substantially lower than the 130.2 G FLOPs of TransFG, the 135.5 G FLOPs of FFVT, and the 162.9 G FLOPs of ACC-ViT. The modest parameter increase to 115.6 M, compared with 87.1 M for the Swin-B backbone, mainly stems from the Prior-Guided Aggregation module and the Topology-Informed Spatial Graph Convolutional Network. These lightweight components enhance feature representation through semantic relation reasoning. Again, we emphasize that the Segment Anything Model is not part of the trainable network architecture; its computational cost is excluded from the above statistics, as it serves only as an external preprocessing tool. This favorable accuracy–efficiency trade-off validates the practicality of STP-Net for large-scale fine-grained recognition tasks.

In summary, the experimental results demonstrate that STP-Net achieves superior recognition performance while maintaining reasonable computational efficiency. The proposed mechanisms for prior-guided part representation, topological relationship reasoning, and semantic–spatial interaction collectively contribute to robust fine-grained visual classification across diverse object structures and visual variations.

### 4.3. Ablation Studies

#### 4.3.1. Analysis of Core Components

To systematically evaluate the effectiveness of each core module and their synergistic effects, comprehensive ablation experiments are conducted on the CUB-200-2011 dataset, with results shown in Table 3. The baseline model employs the original Swin-Base backbone, achieving a Top-1 accuracy of 91.93%. After introducing the Prior-Guided Part Aggregator (PGA) into the baseline, the model accuracy improves to 92.21%, an increase of 0.28% over the baseline. The introduction of PGA improves the classification accuracy by 0.28%, indicating that the semantic prior provides complementary guidance for discriminative part aggregation. Building upon this, the further introduction of the Topology-Informed Semantic Graph Convolution (TIS-GCN) elevates the model performance to 92.42%, yielding an additional 0.21% improvement over the PGA-only model. This demonstrates that relying solely on independent part features is insufficient for adequately modeling the structural dependencies in fine-grained targets, whereas TIS-GCN can dynamically model topological relationships among parts in the implicit semantic space, thereby enhancing the model’s robustness to complex pose variations and structural deformations. Further incorporating the Semantic–Spatial Cross-Attention (SSCA) module, the complete STP-Net ultimately achieves the highest accuracy of 92.68%, representing a cumulative improvement of 0.75% over the baseline. This result validates that SSCA can effectively establish associations between high-level semantic information and spatial structural features, further strengthening the feature representation capability of key discriminative regions through cross-layer semantic interaction.

Overall, with the progressive introduction of PGA, TIS-GCN, and SSCA modules, the model performance exhibits a sustained and stable improvement trend. This not only demonstrates the functional complementarity of each module but also indicates that the proposed prior-guided part modeling, topological relationship reasoning, and semantic–spatial interaction mechanisms can synergistically alleviate background interference and structural instability, thereby obtaining more robust fine-grained visual representations.

#### 4.3.2. Foundation Model Prior Decoupling and Efficiency Analysis

To further analyze the role of foundation model priors at different stages, experiments are conducted on CUB-200-2011 with different SAM usage strategies, with results shown in Table 4. When SAM masks are introduced as spatial priors only during the training phase, the model achieves a Top-1 accuracy of 92.56%, with a single training epoch taking approximately 7 min. This result indicates that the foreground priors provided by SAM can effectively guide part aggregation and topological relationship learning during training, enabling the network to learn stable discriminative structural representations during parameter optimization, thus maintaining strong performance without requiring additional foundation model involvement during inference. In contrast, when SAM is employed in both training and inference phases, the model accuracy slightly decreases to 92.54%, while the single-epoch time increases to approximately 13 min. It should be noted that, in our implementation, SAM masks are generated on-the-fly during each training epoch rather than being pre-computed offline before training. This design choice ensures consistency between the training and inference data processing pipelines. Therefore, the per-epoch training time reported in Table 4 already includes the SAM mask generation cost.

This suggests that continuously introducing foundation model priors during inference may impose redundant constraints on the learned semantic structures, thereby weakening the model’s adaptive representation capability.

#### 4.3.3. Effect of Graph Convolution Depth

**Figure 5:**
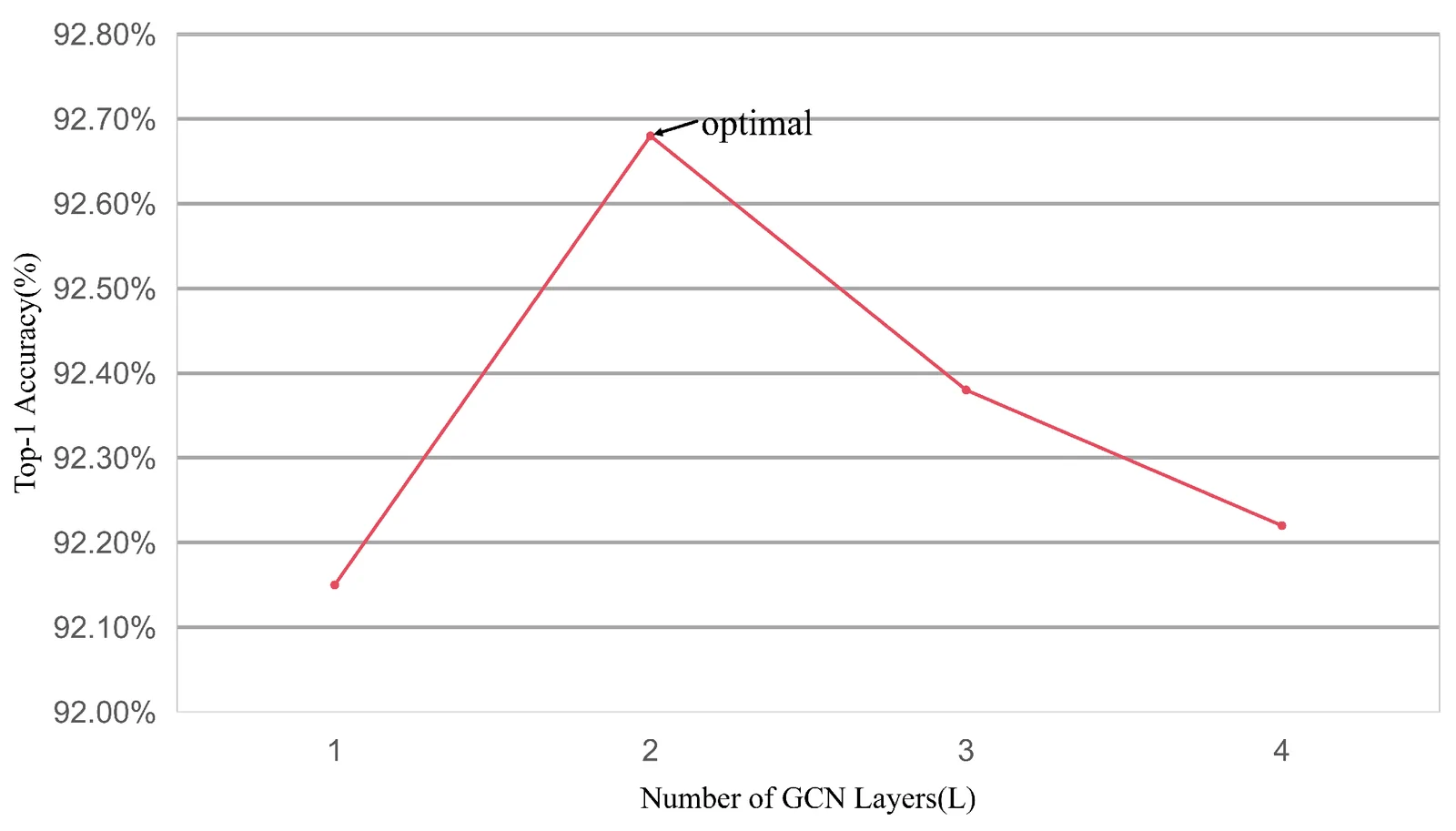
Effect of GCN layer depth on CUB-200-2011.

To evaluate the sensitivity of STP-Net to the graph convolution layer depth in the TIS-GCN module, the impact of different GCN layer numbers on model performance is further analyzed, with experimental results shown in Figure 5. When only a single graph convolution layer is employed, the model achieves a Top-1 accuracy of 92.15%. Due to the limited information propagation range of shallow graph structures, relying on a single neighborhood aggregation is insufficient for adequately modeling complex inter-part semantic dependencies, resulting in certain deficiencies in overall structural representation capability. When the graph convolution depth is increased to two layers, the model performance improves to the highest 92.68%. This result indicates that a two-layer graph convolution can strike a favorable balance between local semantic interaction and global topological modeling, enabling part nodes to accomplish effective information propagation while maintaining strong discriminative representation capability. However, as the graph convolution depth further increases to three and four layers, the model accuracy drops to 92.38% and 92.20%, respectively. This phenomenon indicates that excessively deep graph structures trigger the typical over-smoothing problem, where node features gradually converge after multiple rounds of message propagation, leading to the weakening of discriminative boundaries between different semantic parts and thereby impairing the expressiveness of fine-grained structural features.

#### 4.3.4. Effectiveness Analysis of Auxiliary Deep Supervision

To further evaluate the impact of the auxiliary deep supervision (ADS) strategy on hierarchical feature optimization, model performance under different auxiliary supervision configurations is compared, with experimental results shown in Table 5.

Without any auxiliary supervision, the backbone framework based on PGA and TIS-GCN already achieves a Top-1 accuracy of 92.61%, indicating that the proposed part relationship modeling mechanism itself possesses strong discriminative capability. When an auxiliary classification head is introduced only at the third stage ($S_{3}$), the model accuracy improves to 92.63%. Further extending the auxiliary supervision to the second stage ($S_{2}+S_{3}$) continues to improve the performance to 92.66%.

When auxiliary supervision is simultaneously applied to the first three stages ($S_{1}+S_{2}+S_{3}$), the complete ADS configuration ultimately achieves the best performance of 92.68%. Although this represents only a 0.07% improvement over the no-auxiliary-supervision baseline, such a stable incremental gain remains significant for fine-grained visual classification tasks that are already approaching performance saturation.

The experimental results demonstrate that as auxiliary supervision progressively extends toward shallower network layers, the model performance exhibits a consistent improvement trend. This indicates that ADS can effectively alleviate the insufficient gradient propagation in deep Transformers, providing more stable optimization signals for shallow feature learning and thereby promoting the full learning of low-level detail features such as edges and textures.

#### 4.3.5. Effectiveness of Global-Context Adaptive Gating

To validate the effectiveness of the Global-Context Adaptive Gating (GCAG) module in multi-scale feature fusion, comparative experiments are conducted with traditional static fusion strategies, with results shown in Table 6.

When global average pooling (GAP) is employed for multi-scale feature fusion, the model achieves a Top-1 accuracy of 92.52%; further using global max pooling (GMP) improves the accuracy to 92.55%. Although max pooling can better highlight local salient responses, both static pooling approaches inherently assume that features at different levels possess fixed and equal importance, making it difficult to adaptively adjust the contribution of different scale features based on the structural variations of input images.

In contrast, the proposed GCAG module achieves the highest accuracy of 92.68%, further improving by 0.13% over GMP. This performance gain indicates that GCAG can more effectively model the complementary relationships among multi-scale semantics. Specifically, GCAG first obtains an overall semantic description through global context aggregation and then adaptively generates channel weights through the gating mechanism, thereby dynamically regulating the contribution proportions of features at different stages. This process not only effectively suppresses redundant information but also highlights the most discriminative level-specific semantic representations for the current input.

Overall, the experimental results demonstrate that, compared to traditional unweighted static fusion approaches, the global context-based adaptive gating mechanism is more suitable for multi-scale feature integration in fine-grained visual classification tasks, capable of further enhancing the model’s expressiveness for complex structural details.

### 4.4. Visualization Analysis

#### 4.4.1. Multi-Stage Feature Response Visualization

**Figure 6:**
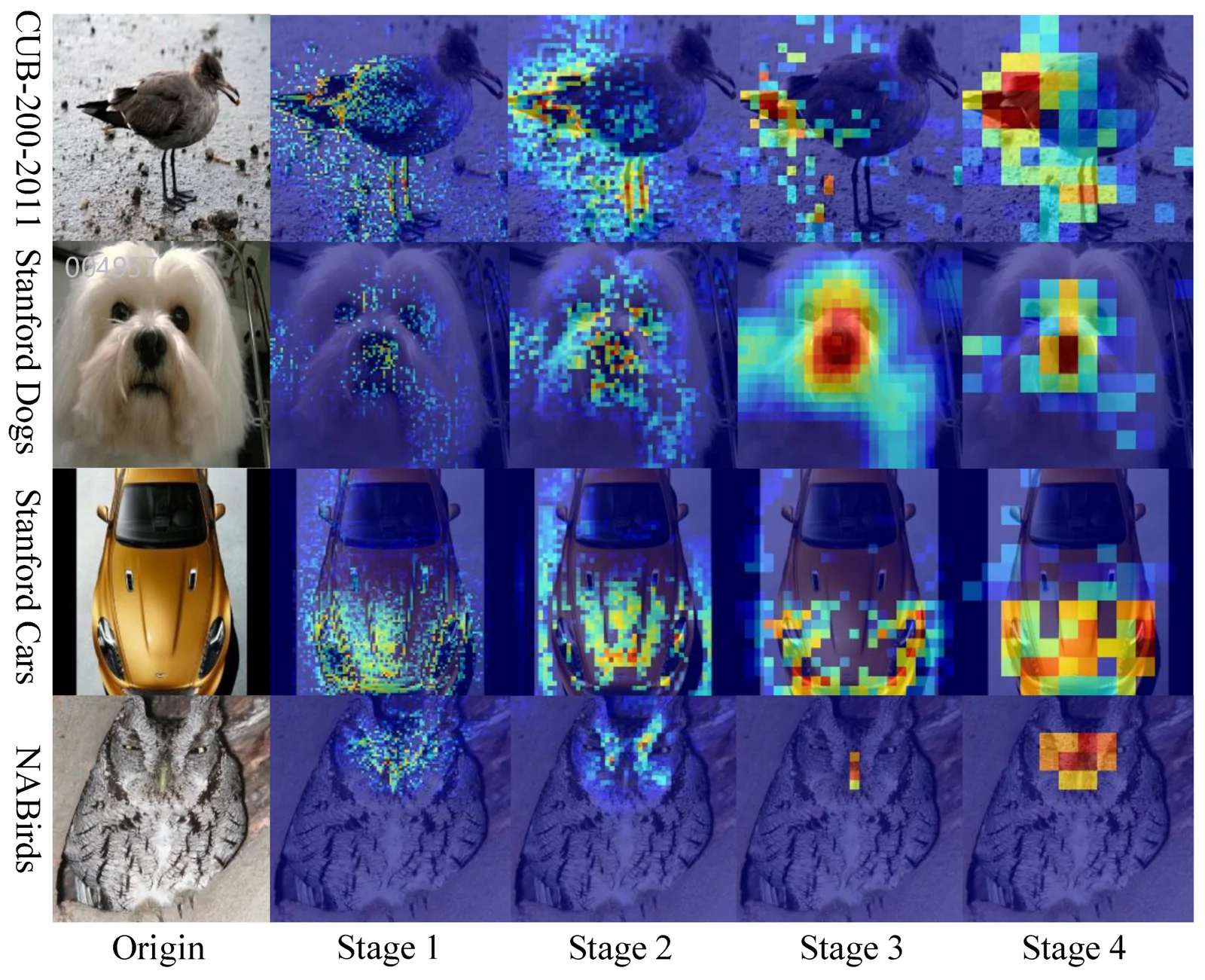
Visualization of feature responses at different stages of STP-Net.

To further analyze the feature evolution patterns of STP-Net during hierarchical feature learning, the response results at different stage outputs are visualized, as shown in Figure 6. From Stage 1 to Stage 4, the model’s attention regions exhibit a progressive evolution from dispersed to concentrated, and from low-level textures to high-level semantics.

In Stage 1, the network primarily focuses on low-level visual information such as target contours and texture edges, with response regions being relatively dispersed and subject to partial background interference. As the network enters Stage 2, benefiting from the foreground prior constraints introduced by the PGA module, background noise is effectively suppressed, and the model begins to concentrate its attention on the target region. Upon entering Stage 3, TIS-GCN models the relationships among semantic parts, enabling the model to further mine structurally meaningful discriminative information. For example, in bird samples, the focus gradually concentrates on the head and beak regions; in dog samples, attention is primarily directed toward the eyes and nose regions; in vehicle samples, the main focus is on the headlights and front fascia structures. Finally, in Stage 4, the attention further converges onto the key parts most critical for category discrimination, forming compact and stable response patterns.

The above results demonstrate that STP-Net can progressively filter out redundant information through multi-stage progressive modeling and continuously strengthen the feature representation of key discriminative regions, achieving the transition from local texture representation to high-level semantic structural representation.

#### 4.4.2. Grad-CAM Discriminative Region Visualization

**Figure 7:**
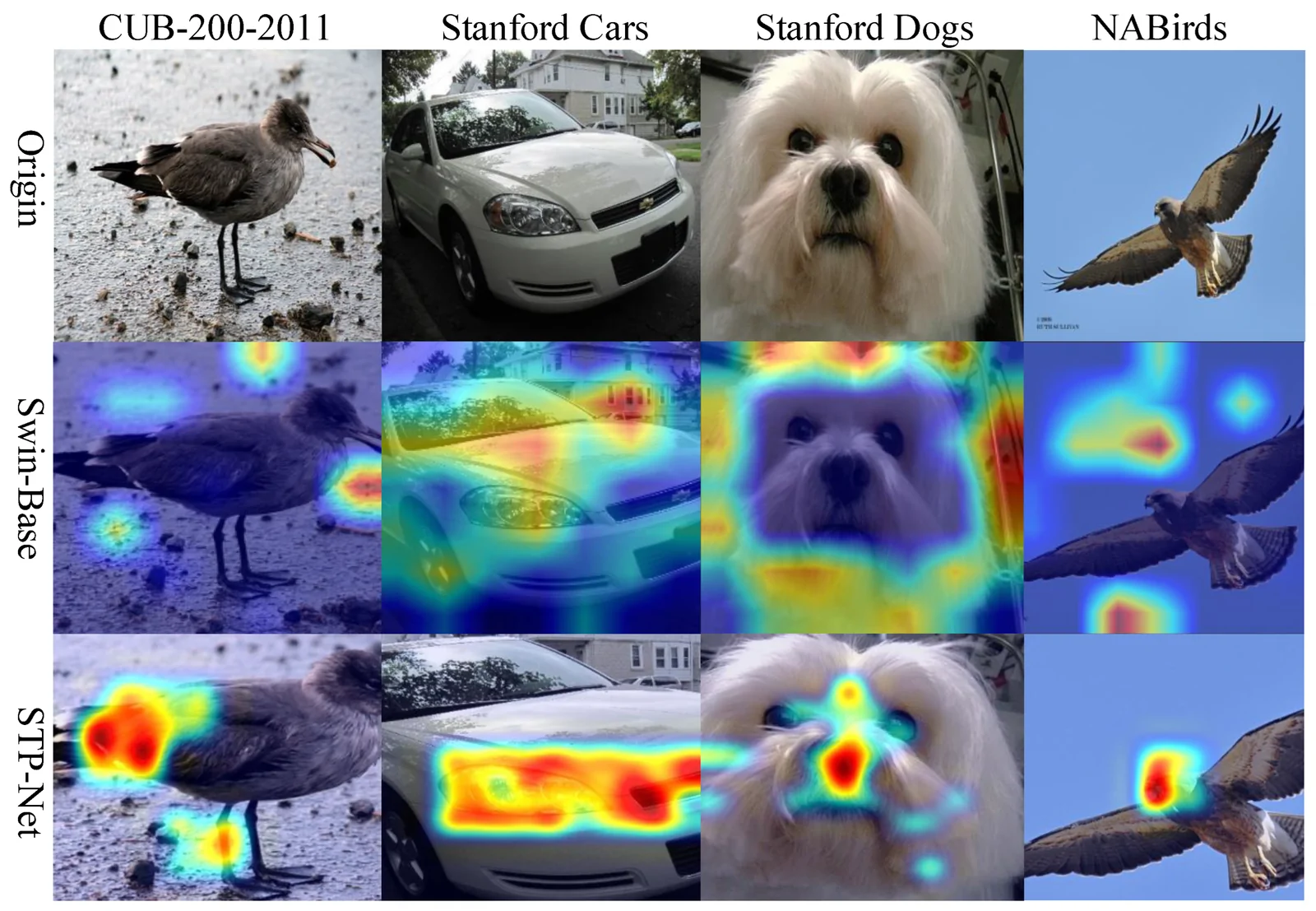
Grad-CAM visualization comparison between Swin-Base and STP-Net.

To validate STP-Net’s capability in localizing discriminative regions and its interpretability, Grad-CAM is employed to visualize the model’s final prediction results, with a comparison against Swin-Base, as shown in Figure 7.

It can be observed that while Swin-Base can roughly localize the target, its response regions are relatively dispersed, with partial attention still falling on background or non-critical regions. For instance, in bird samples, the model simultaneously attends to the surrounding environment of the target; in vehicle samples, the heatmap coverage is extensive, making it difficult to highlight the truly discriminative structural details. This indicates that relying solely on the self-attention mechanism, the model is susceptible to the influence of background information and redundant features.

In contrast, the heatmaps generated by STP-Net are more concentrated with clearer boundaries. Across samples from different datasets, the model can accurately focus on key parts with fine-grained discriminative significance, such as bird heads, dog faces, and vehicle front fascias, while the responses to background regions are markedly diminished. This demonstrates that the PGA module effectively enhances the learning capability for foreground semantic regions, TIS-GCN further strengthens the structural relationship modeling among parts, and SSCA remaps the learned high-level semantic relationships back into spatial features, thereby enhancing the response intensity of key regions.

Overall, the Grad-CAM visualization results demonstrate that STP-Net not only learns more accurate discriminative features but also effectively suppresses background interference, making the model’s decision basis more aligned with the true semantic regions of the target, further validating the effectiveness and interpretability of the proposed network architecture.

## 5. Conclusions

To address the challenges of severe background interference, difficulty in accurately extracting local discriminative information, and insufficient modeling of structural relationships among parts in fine-grained visual classification, this paper proposes a Semantic Topology Multi-Scale Part Network (STP-Net). Built upon hierarchical multi-scale feature representations, the method introduces a Prior-Guided Part Aggregator (PGA) that leverages foreground priors provided by foundation models to guide the network toward discriminative target regions while suppressing background noise. A Topology-Informed Semantic Graph Convolution (TIS-GCN) is designed to dynamically construct inter-part relationships in an implicit semantic space, achieving robust structural modeling against pose variations and non-rigid deformations. Furthermore, a Semantic–Spatial Cross-Attention (SSCA) mechanism is employed to establish effective interaction between semantic relationships and spatial features, and combined with a Global-Context Adaptive Gating (GCAG) module for multi-scale feature fusion, thereby obtaining more robust and discriminative feature representations. Experimental results on four public fine-grained visual classification benchmarks, namely CUB-200-2011, Stanford Cars, Stanford Dogs, and NABirds, demonstrate that the proposed method achieves superior and stable classification performance. Ablation studies validate the effectiveness of each core module, and visualization analyses further confirm that the model can more accurately localize key discriminative regions and learn structure-aware semantic representations. Overall, by integrating foundation model priors, multi-scale part modeling, and semantic topological relationship learning, STP-Net effectively enhances the feature representation capability and classification performance of fine-grained targets, providing an effective solution for structured representation learning in fine-grained visual classification.

## Figures and Tables

**Table 1:** Dataset splits and learning rates.

Dataset	Categories	Training	Testing	Learning Rate
CUB-200-2011 [31]	200	5994	5794	$8\times 10^{-3}$
Stanford Cars [32]	196	8144	8041	$8\times 10^{-4}$
Stanford Dogs [33]	120	12,000	8580	$8\times 10^{-5}$
NABirds [34]	555	23,929	24,633	$16\times 10^{-3}$

The numbers of training and testing samples follow the official dataset splits.

**Table 2:** Performance comparison of various methods on FGVC benchmark datasets.

Methods	Backbone	CUB-200-2011	Stanford Cars	Stanford Dogs	NABirds	FLOPs	Params
Cross-X [12]	ResNet-50	87.7	94.6	88.9	86.2	-	-
MSHQP [14]	ResNet-50	89.0	-	90.4	-	-	-
P2P-Net [36]	ResNet-50	90.2	**95.4**	-	-	-	-
FDL [37]	DenseNet-161	89.1	94.0	84.9	-	-	-
API-Net [11]	DenseNet-161	90.0	95.3	90.3	88.1	-	-
CAMF [38]	Swin-B	91.2	95.3	92.8	90.9	-	-
FFVT [39]	ViT-B-16	91.6	-	91.5	-	135.5 G	86.4 M
TransFG [15]	ViT-B-16	91.7	94.8	92.3	90.8	130.2 G	86.4 M
LDH-ViT [17]	ViT-B-16	91.7	93.1	93.1	90.2	-	-
IELT [16]	ViT-B-16	91.8	-	91.8	90.8	73.2 G	93.5 M
DAVT [18]	ViT-B-16	91.8	-	91.0	-	134.5 G	86.4 M
MP-FGVC [21]	ViT-B-16	91.8	-	91.0	91.0	-	-
ACC-ViT [20]	ViT-B-16	91.8	-	92.9	91.4	162.9 G	87.0 M
STP-Net (Ours)	Swin-B	**92.7 ± 0.08**	94.9 ± 0.06	**95.2 ± 0.05**	**92.3 ± 0.10**	49.5 G	115.6 M

**Note:** Values in bold indicate the bestresults in each column.

**Table 3:** Ablation study of each module on the CUB-200-2011 dataset.

Module			Accuracy (%)	Improv. (%)
PGA	TIS-GCN	SSCA		
-	-	-	91.93 ± 0.09	-
✓	-	-	92.21 ± 0.11	+0.28
✓	✓	-	92.42 ± 0.10	+0.49
✓	✓	✓	92.68 ± 0.08	+0.75

**Note:** ✓ indicates that the corresponding module is used.

**Table 4:** Impact of different SAM usage strategies on model performance and computational efficiency.

Method	Training	Inference	Acc. (%)	Time (min/Epoch)
SAM	✓	×	92.56	7
SAM	✓	✓	92.54	13

**Note:** ✓ indicates that SAM is used; × indicates that SAM is not used.

**Table 5:** Refined ablation of the ADS strategy on the CUB-200-2011 dataset. $S_{i}$ denotes the auxiliary classification head applied to the *i*-th stage feature map.

Configuration	ADS Placement	Accuracy (%)	Improv. (%)
Baseline(STP-Net *w*/*o* ADS)	None	92.61	-
Variant 1	$S_{3}$	92.63 ± 0.04	+0.02
Variant 2	$S_{2}+S_{3}$	92.66 ± 0.02	+0.05
STP-Net (Full)	$S_{1}+S_{2}+S_{3}$	92.68 ± 0.05	+0.07

**Table 6:** Ablation study of feature fusion strategies after multi-scale concatenation on the CUB-200-2011 dataset.

Fusion Strategy	Top-1 Accuracy (%)	Improv. (%)
Average Pooling	92.52 ± 0.07	-
Max Pooling	92.55 ± 0.10	+0.03
GCAG (Ours)	92.68 ± 0.09	+0.16

## Data Availability

The datasets used in this study are publicly available benchmark datasets and can be obtained from their respective official sources.

## Abbreviations

The following abbreviations are used in this manuscript:

FGVC	Fine-Grained Visual Classification
PGA	Prior-Guided Part Aggregator
TIS-GCN	Topology-Informed Semantic Graph Convolutional Network
SSCA	Semantic–Spatial Cross-Attention
GCAG	Global-Context Adaptive Gating
SAM	Segment Anything Model
ADS	Auxiliary Deep Supervision
GAC	Gradient-Attention Consistency
GCN	Graph Convolutional Network

## References

[1] Al Amin M., Jung H.Y. (2026). Fine-grained Visual Classification: A Survey of Methods, Architectures, and Emerging Trends. IEEE Access.

[2] Chou P.Y., Kao Y.Y., Lin C.H. (2023). Fine-grained visual classification with high-temperature refinement and background suppression. arXiv.

[3] Sun H., He X., Peng Y. Sim-trans: Structure information modeling transformer for fine-grained visual categorization. Proceedings of the 30th ACM International Conference on Multimedia.

[4] Kirillov A., Mintun E., Ravi N., Mao H., Rolland C., Gustafson L., Xiao T., Whitehead S., Berg A.C., Lo W.Y. Segment Anything. Proceedings of the IEEE/CVF International Conference on Computer Vision (ICCV).

[5] Zhang N., Donahue J., Girshick R., Darrell T. Part-based R-CNNs for fine-grained category detection. Proceedings of the European Conference on Computer Vision.

[6] Wei X.S., Xie C.W., Wu J., Shen C. (2018). Mask-CNN: Localizing parts and selecting descriptors for fine-grained bird species categorization. Pattern Recognit..

[7] Branson S., Van Horn G., Belongie S., Perona P. (2014). Bird species categorization using pose normalized deep convolutional nets. arXiv.

[8] Fu J., Zheng H., Mei T. Look closer to see better: Recurrent attention convolutional neural network for fine-grained image recognition. Proceedings of the IEEE Conference on Computer Vision and Pattern Recognition.

[9] Yang Z., Luo T., Wang D., Hu Z., Gao J., Wang L. Learning to navigate for fine-grained classification. Proceedings of the European Conference on Computer Vision (ECCV).

[10] Chen Y., Bai Y., Zhang W., Mei T. Destruction and construction learning for fine-grained image recognition. Proceedings of the IEEE/CVF Conference on Computer Vision and Pattern Recognition.

[11] Zhuang P., Wang Y., Qiao Y. Learning attentive pairwise interaction for fine-grained classification. Proceedings of the AAAI Conference on Artificial Intelligence.

[12] Luo W., Yang X., Mo X., Lu Y., Davis L.S., Li J., Lim S.N. Cross-x learning for fine-grained visual categorization. Proceedings of the IEEE/CVF International Conference on Computer Vision.

[13] Du R., Chang D., Bhunia A.K., Xie J., Ma Z., Song Y.Z., Guo J. Fine-grained visual classification via progressive multi-granularity training of jigsaw patches. Proceedings of the European Conference on Computer Vision.

[14] Tan M., Yuan F., Yu J., Wang G., Gu X. (2022). Fine-grained image classification via multi-scale selective hierarchical biquadratic pooling. ACM Trans. Multimed. Comput. Commun. Appl..

[15] He J., Chen J.N., Liu S., Kortylewski A., Yang C., Bai Y., Wang C. (2022). TransFG: A transformer architecture for fine-grained recognition. Proc. AAAI Conf. Artif. Intell. Virtual.

[16] Xu Q., Wang J., Jiang B., Luo B. (2023). Fine-grained visual classification via internal ensemble learning transformer. IEEE Trans. Multimed..

[17] Shi Y., Hong Q., Yan Y., Li J. (2025). LDH-ViT: Fine-grained visual classification through local concealment and feature selection. Pattern Recognit..

[18] Hu C., Zhu L., Qiu W., Wu W. (2022). Data augmentation vision transformer for fine-grained image classification. arXiv.

[19] Chen H., Zhang H., Liu C., An J., Gao Z., Qiu J. (2024). FET-FGVC: Feature-enhanced transformer for fine-grained visual classification. Pattern Recognit..

[20] Zhang Z.C., Chen Z.D., Wang Y., Luo X., Xu X.S. (2024). A vision transformer for fine-grained classification by reducing noise and enhancing discriminative information. Pattern Recognit..

[21] Jiang X., Tang H., Gao J., Du X., He S., Li Z. Delving into multimodal prompting for fine-grained visual classification. Proceedings of the AAAI Conference on Artificial Intelligence.

[22] Zhu L., Chen T., Yin J., See S., Liu J. Learning gabor texture features for fine-grained recognition. Proceedings of the IEEE/CVF International Conference on Computer Vision.

[23] Yu Y., Wei W., Zhao C., Qian J., Chen E. (2026). Structural feature enhanced transformer for fine-grained image recognition. Pattern Recognit..

[24] Wang Z.G., Chen S.B., Luo B. (2025). Background noise suppression for advanced fine-grained visual classification. Neurocomputing.

[25] Wang S., Wang Z., Li H., Chang J., Ouyang W., Tian Q. (2024). Accurate fine-grained object recognition with structure-driven relation graph networks. Int. J. Comput. Vis..

[26] Zhao Y., Yan K., Huang F., Li J. Graph-based high-order relation discovery for fine-grained recognition. Proceedings of the IEEE/CVF Conference on Computer Vision and Pattern Recognition.

[27] Wang D., Wang Q., Min W., Gai D., Han Q., Li L., Geng Y. (2024). SAM-driven MAE pre-training and background-aware meta-learning for unsupervised vehicle re-identification. Comput. Vis. Media.

[28] Yin S., Peng Y. Part-Aware CLIP: Enhancing Fine-Grained Understanding with Part-level Descriptions. OpenReview 2026, c1VgleBVlw. https://openreview.net/forum?id=c1VgleBVlw.

[29] Amir S., Gandelsman Y., Bagon S., Dekel T. (2021). Deep vit features as dense visual descriptors. arXiv.

[30] Wang J., Xu Q., Jiang B., Luo B., Tang J. (2024). Multi-granularity part sampling attention for fine-grained visual classification. IEEE Trans. Image Process..

[31] Wah C., Branson S., Welinder P., Perona P., Belongie S. (2011). The Caltech-UCSD Birds-200-2011 Dataset.

[32] Krause J., Stark M., Deng J., Fei-Fei L. 3D object representations for fine-grained categorization. Proceedings of the IEEE International Conference on Computer Vision Workshops.

[33] Khosla A., Jayadevaprakash N., Yao B., Li F.F. Novel dataset for fine-grained image categorization: Stanford dogs. Proceedings of the CVPR Workshop on Fine-Grained Visual Categorization (FGVC).

[34] Van Horn G., Branson S., Farrell R., Haber S., Barry J., Ipeirotis P., Perona P., Belongie S. Building a bird recognition app and large scale dataset with citizen scientists: The fine print in fine-grained dataset collection. Proceedings of the IEEE Conference on Computer Vision and Pattern Recognition (CVPR).

[35] Liu Z., Lin Y., Cao Y., Hu H., Wei Y., Zhang Z., Lin S., Guo B. Swin Transformer: Hierarchical Vision Transformer Using Shifted Windows. Proceedings of the IEEE/CVF International Conference on Computer Vision (ICCV).

[36] Yang X., Wang Y., Chen K., Xu Y., Tian Y. Fine-grained object classification via self-supervised pose alignment. Proceedings of the IEEE/CVF Conference on Computer Vision and Pattern Recognition.

[37] Liu C., Xie H., Zha Z.J., Ma L., Yu L., Zhang Y. Filtration and distillation: Enhancing region attention for fine-grained visual categorization. Proceedings of the AAAI Conference on Artificial Intelligence.

[38] Miao Z., Zhao X., Wang J., Li Y., Li H. (2021). Complemental attention multi-feature fusion network for fine-grained classification. IEEE Signal Process. Lett..

[39] Wang J., Yu X., Gao Y. (2021). Feature fusion vision transformer for fine-grained visual categorization. arXiv.

